# Potential Target Site for Inhibitors in MLS_B_ Antibiotic Resistance

**DOI:** 10.3390/antibiotics10030264

**Published:** 2021-03-05

**Authors:** Hak Jin Lee, Seong Tae Jhang, Hyung Jong Jin

**Affiliations:** 1 Department of Life Science, Korea University Graduate School, Seoul 02841, Korea; stork526@naver.com; 2 Department of Bioscience and Biotechnology, The University of Suwon, Whasung City 18323, Korea; 3 College of Information Technology, The University of Suwon, Whasung City 18323, Korea; stjhang@suwon.ac.kr

**Keywords:** MLS_B_ antibiotic resistance, Erm proteins, methylation, mutation, target site for inhibitor development

## Abstract

Macrolide–lincosamide–streptogramin B antibiotic resistance occurs through the action of erythromycin ribosome methylation (Erm) family proteins, causing problems due to their prevalence and high minimal inhibitory concentration, and feasibilities have been sought to develop inhibitors. Erms exhibit high conservation next to the N-terminal end region (NTER) as in ErmS, 64SQNF67. Side chains of homologous S, Q and F in ErmC’ are surface-exposed, located closely together and exhibit intrinsic flexibility; these residues form a motif X. In S64 mutations, S64G, S64A and S64C exhibited 71%, 21% and 20% activity compared to the wild-type, respectively, conferring cell resistance. However, mutants harboring larger side chains did not confer resistance and retain the methylation activity in vitro. All mutants of Q65, Q65N, Q65E, Q65R, and Q65H lost their methyl group transferring activity in vivo and in vitro. At position F67, a size reduction of side-chain (F67A) or a positive charge (F67H) greatly reduced the activity to about 4% whereas F67L with a small size reduction caused a moderate loss, more than half of the activity. The increased size by F67Y and F67W reduced the activity by about 75%. In addition to stabilization of the cofactor, these amino acids could interact with substrate RNA near the methylatable adenine presumably to be catalytically well oriented with the SAM (S-adenosyl-L-methionine). These amino acids together with the NTER beside them could serve as unique potential inhibitor development sites. This region constitutes a divergent element due to the NTER which has variable length and distinct amino acids context in each Erm. The NTER or part of it plays critical roles in selective recognition of substrate RNA by Erms and this presumed target site might assume distinct local structure by induced conformational change with binding to substrate RNA and SAM, and contribute to the specific recognition of substrate RNA.

## 1. Introduction

Microorganisms protect themselves against the action of antibiotics through three major mechanisms [1]: (1) they modify the target site for antibiotics that related antibiotics could not work on [2]; (2) the antibiotic access to the targets is prevented [3]; (3) the chemical modification of antibiotics could be carried out to inactivate them [4]. For macrolide–lincosamide–streptogramin B (MLS_B_) antibiotics, all three resistance mechanisms occur as well [5,6] but target site modification is the most problematic due to its prevalence in resistant pathogens and high level of minimal inhibitory concentration value that could hardly be attained and overcome by the administration of available antibiotics. Because MLS_B_ antibiotics have quite different chemical structures but share a common resistance mechanism, the exocyclic N^6^-methylation of a specific adenine residue (A2058 in 23S rRNA, *Escherichia coli* coordinate), they are categorized into one superfamily of antibiotics. This target site modification is carried out by a family of enzymes named erythromycin ribosome methylation (Erm). Therefore, it has long been considered that the specific inhibitor(s) to the member(s) of the Erm family could help overcome antibiotic resistance to attain the proper therapeutic goals. Several lines of strategies to develop inhibitors have been proposed and executed. In the beginning, the enzymatic activity itself was used for screening and detecting compounds possessing a potential inhibitory activity [7]. Following this work, nuclear magnetic resonance-based screening and chemical synthesis based on that, could lead to novel compounds that bind to the *S*-adenosyl-L-methionine (SAM) binding sites of ErmC’ [8] and ErmB [9] and demonstrated that the resistance could be overcome using them [10]. The virtual screening of compounds based on the structure of Erm protein and their characterization by in vitro potency [11], and proposals to design the inhibitors based on the minimal substrate features [12] have been reported as well. Although the SAM binding mode is highly conserved among different methyltransferases (MTases), it was proposed to take advantage of the distinct conformation characteristic of ErmC’ for designing an inhibitor and further modify it to displace the structurally conserved waters observed in the SAM binding site of ErmC’ and include the structural mimics of the transition state to improve the potency [13]. Furthermore, the recent elucidation of the functions of the unconventional carbon-oxygen (CH^…^O) hydrogen bond between the transferrable methyl group of SAM and oxygen from certain active site residue(s) of the enzyme, which contribute to high-affinity SAM binding, transition state stabilization and limitation of the motion of the SAM methyl group to maintain the alignment of the methyl group for optimal transfer geometry, could provide the two distinct strategies for the specific inhibitor development of MTases: (1) to design the ligands to act as CH^…^O hydrogen bond acceptors instead of SAM or (2) to mimic SAM CH^…^O hydrogen-bond donors using cofactor or transition state analogs [14]. No such CH^…^O hydrogen bond has not been reported in any member of Erm proteins, whereas for one member in the same class of MTase, (L-isoaspartyl O-methyltransferase), such hydrogen bond has been reported. Therefore, such a CH^…^O hydrogen bond could be possible even in Erm proteins, and those strategies might apply to the development of inhibitors for Erm proteins. To avoid possible adverse effects by acting on the various SAM-dependent MTases through conserved SAM binding pockets, blocking the unique interaction sites of Erm protein with RNA substrate has been proposed for developing inhibitors that could improve selectivity and might attain greater potency [15]. KsgA/Dim1 is currently known as the only one protein family with a homologous relationship to Erm protein [16]. Using and modifying KsgA (BsKsgA) from *Bacillus subtilis* based on structural and phylogenetic analysis, specificity could be switched from that of KsgA, dimethylation at two adjacent adenines A1518 and A1519 in the 3′-end of 16S rRNA, to that of Erm, A2058 of 23S rRNA by either swapping the two loops alone or C-terminal domain together or two loops but truncating C-terminal domain. From these observations, the target region of protein-RNA interaction for inhibitor development in Erm proteins could be narrowed down. Two supposed RNA binding sites (designated as loops 1 and 12) exhibiting the potential for a partial shift of substrate specificity from KsgA to Erm by swapping between KsgA and ErmC’ were suggested for the prospective inhibitor development target without probable cross-reactivity with other SAM-dependent MTases sharing the similar S_N_2 reaction mechanism [17]. Loop 1 might be the N-terminal end region (NTER) or part of it immediately 5′ to the shortest motif X (GQNF, see below), forming one of the divergent elements in Erm proteins and loop 12 is the shortened version of loop 12 of BsKsgA located between helices α7 and α8 because in ErmC’ and ErmB, only two amino acids constitute that region. 

Among the motifs (I-X) that could be identified in DNA MTases to yield C5-methylcytosine, nine motifs, except motif IX, could be located in Erm proteins [8]. Each motif could be assigned its specific function such that motifs I to III, V, and X could be involved in SAM binding and motifs IV, VI and VIII could be related to catalysis. However, in many cases, each motif might not solely dedicate itself to the assigned role, that is, they often play multiple roles. Although the P-loop containing the DPPY motif in motif IV forms the active site with motifs IV and VIII, in M. *Hha*I, it contributes to SAM binding using the peptide backbone of the P-loop. Motif V is involved in both binding SAM by making van der Waals contacts and forming the active site with motifs IV, VI, VIII and VII, and so on [18]. In ErmC’ and ErmB, the motif X sequence is located near the active site and presumably, each amino acid might perform more than just one role. The exact location and range of motif X in Erm proteins are somewhat different depending on the reports [17,19,20]. However, in that motif, some consensus G(S/T)-Q-N(H)-F(L, Y) in which Q is highly conserved among almost all members of the families of Erm, and another sequence might be altered to amino acid(s) in parenthesis, could be identified with Erm families and some other slight variation in that consensus could be found with KsgA/Dim1 (see below). Although these amino acids were observed to be in immediate adjacency to SAM, this region was exhibiting different conformations when ErmC’ bound to different ligands [SAM, S-adenosyl-L-homocysteine (SAH) and sinefungin), presumably suggesting intrinsic flexibility [13] and probable functional importance. When targeting specificity of BsKsgA was attempted to be switched to that of Erm, swapping with ErmC’ of unstructured sequence from N-terminal up to the shortest motif X and loop sequence interfacing catalytic domain and C-terminal domain brought about desired change dramatically and successfully. Among these regions, the unstructured region preceding the shortest motif X should reside near the active site. However, the sequence and length of this region vary among the Erm family members. Therefore, we assumed that other sequence(s) besides this region could play additional important roles in the specific and/or selective recognition of substrate and/or proper positioning target adenine in the active site. This could be a well-conserved sequence such as (G/S/T)-Q-(N/H)-(F/L/Y), in a relaxed form. In this regard, the mutation of each amino acid in this sequence employing ErmS, one of the dimethyltransferase from *Streptomyces fradiae*, a tylosin producer [21] was carried out. The effects of each mutation were characterized in vitro and in the cell. 

## 2. Results

### 2.1. Sequence Alignment of Erm and KsgA/Dim1

**Figure 1:**
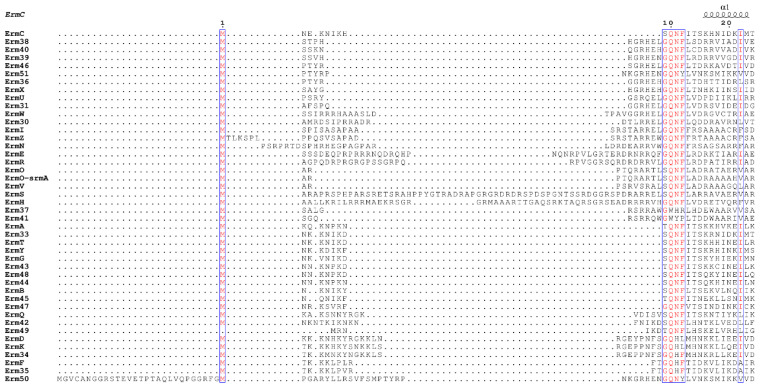
Alignment of the NTER and the shortest motif X of erythromycin ribosome methylation (Erm) proteins. The region of the 44 Erm proteins known to date was aligned, which contains the NTER, the shortest motif X (boxed), and first α-helix. The NTER encompasses the N-terminal end up to the shortest motif X represented by GQNF. There is no conserved sequence detected in the NTER whereas high conservation could be found in the shortest motif X. There is a wide range of variation in the length of the NTER among Erm proteins. When loops 1 and 12 of BsKsgA were swapped with ErmC’, substrate specificity could be switched partially from that of KsgA to Erm’s specificity. In Erm proteins, loop 1 could be the NTER or part of it depending on the individual Erm protein, but it could be located immediately adjacent to the shortest motif X [17]. Therefore, loop 1 could contribute to the selective and specific recognition of the substrate for Erm. For the proper function of Erm proteins, the relatively strict and/or absolute requirement on the identity of the surface-exposed side chain of S–Q–F in the shortest motif X should be met. This region encompassing the NTER and the shortest motif X are intrinsically flexible. Furthermore, this region’s unique conformational change could be expected with the binding of SAM and the substrate. Therefore, it could be the potential target site for the inhibitor development. See Discussion for details. The alignment picture was prepared with ESPript 3.0 [33].

From the Erm family, a total of 44 sequences were aligned, where 41 of them could be retrieved from CARD (comprehensive antibiotic resistance database; [22]) and ErmI [23,24], Erm50 [25] and Erm51 [26] were added to them. To the authors’ knowledge, these are all Erm proteins reported at the time of submission of the paper. The length of the starting amino acid (M) to that just before the shortest motif X (NTER) varied from the longest of ErmS (63 amino acids) to the shortest of Erm49 (6 amino acids), and any consensus could not be detected clearly among them. However, immediately after that, conspicuous conservation of amino acids could be recognized in a relaxed form, described as the shortest motif X (Figure 1). In this motif, the first amino acid could be represented as G in most Erm proteins: 26 including Erm37 and Erm41 of 44 Erms. S could be observed in 14 Erm proteins and T also in 4 Erms. In the second position, Q is completely conserved, except Erm37 and Erm41, which do not harbor C-terminal domain alike and where Q is switched to W. Erm37 exhibits promiscuous methylating activity at adjacent positions in the vicinity of A2058 [27], whereas Erm38 is an A2058-specific dimethylase, the activity of which is found to be lethargic, and most rRNAs being either monomethylated or unmethylated [28]. At the third position, N could be located in 37 Erms, but change to H could be detected in 6 Erms; for Erm41, Y could be there instead of N. For the last position, F is conserved in 38 Erms, except 6 Erms. Whereas in two of them (ErmD and ErmK) L is present in place of F, the remaining two Erms, Erm50 and Erm51 contain Y. R and P take that place in Erm37 and Erm41, respectively. Therefore, the shortest motif X mutated in this study is well-conserved in Erm proteins, including monomethyltransferases, such as ErmN [29], ErmO [30], ErmU [31] and Erm30 [32], except for Erm37 and Erm41. A limited number of KsgA/DimI and Archaea homologs were BLAST-searched for detecting the variation in the length of the NTER 5′ to the shortest motif X and amino acid identity of motif X using KsgA sequences of *Algoriphagus machipongonensis*, *B. subtilis*, *Bacteroides fragilis* and *Bifidobacterium longum* as queries. The length of the NTER varies from 10 to 30 amino acids, no conservation in amino acid sequence in this region could be detected and G-Q-(H/N)-(F/W), the relaxed form of conservation in motif X could be observed.

### 2.2. Expression of Mutant Proteins in E. coli

**Figure 2:**
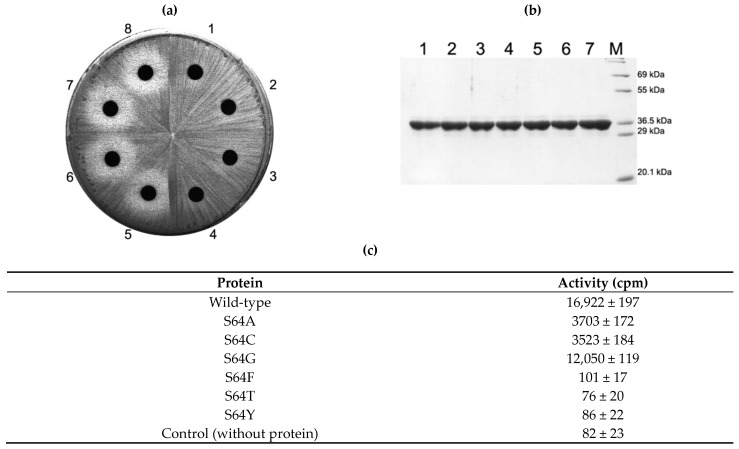
In vivo and in vitro analysis of S64 mutant protein activity. S64A, S64C, S64G, S64F, S64T and S64Y mutant proteins were overexpressed in *E. coli* BL21(DE3). Each protein could be expressed in a similar amount in the cell and purified to a near homogeneity (**b**). In the cell, mutant proteins whose side chains are smaller than the wild type (serine) conferred resistance to erythromycin, whereas mutants with a larger side chain than serine failed to do so (**a**). These observations could be confirmed with the in vitro activity assay with purified proteins (**c**). See Discussion for details. 1, *E. coli* BL21(DE3) expressing the wild-type protein in (**a**) and its purified protein in (**b**); 2, cells expressing S64A mutant and its purified protein; 3, cells with S64C and its purified protein; 4, cells with S64G and its purified protein; 5, cells with S64F and its protein; 6, cells with S64F and its protein; 7, cells with S64Y and its protein; 8, cells containing only empty vector, pET23b; M, molecular weight marker.

The highly conserved amino acids S64, Q65 and F67 were mutated to related amino acids such as G, A, C, T, F, and Y (for S64), E, N, R, and H (for Q65), and A, H, L, W and Y (for F67). All mutant proteins could be overexpressed mainly as a soluble form in a similar amount to the wild-type protein (Figure 2b, Figures 4b and 5b; 126 mg/L culture) [34]. The behavior in the affinity column was observed to be quite similar. The side chains of these amino acids point outward to the solvent and these amino acids belong to the intrinsically flexible region of the protein (see below). These might suggest that mutations introduced at S64, Q65 and F67 in this study might not induce any structural perturbation. Based on this observation, the effects of the introduced mutation on the activity of ErmS in cells and in vitro was investigated as follows.

### 2.3. Activity of S64 Mutants

**Figure 3:**
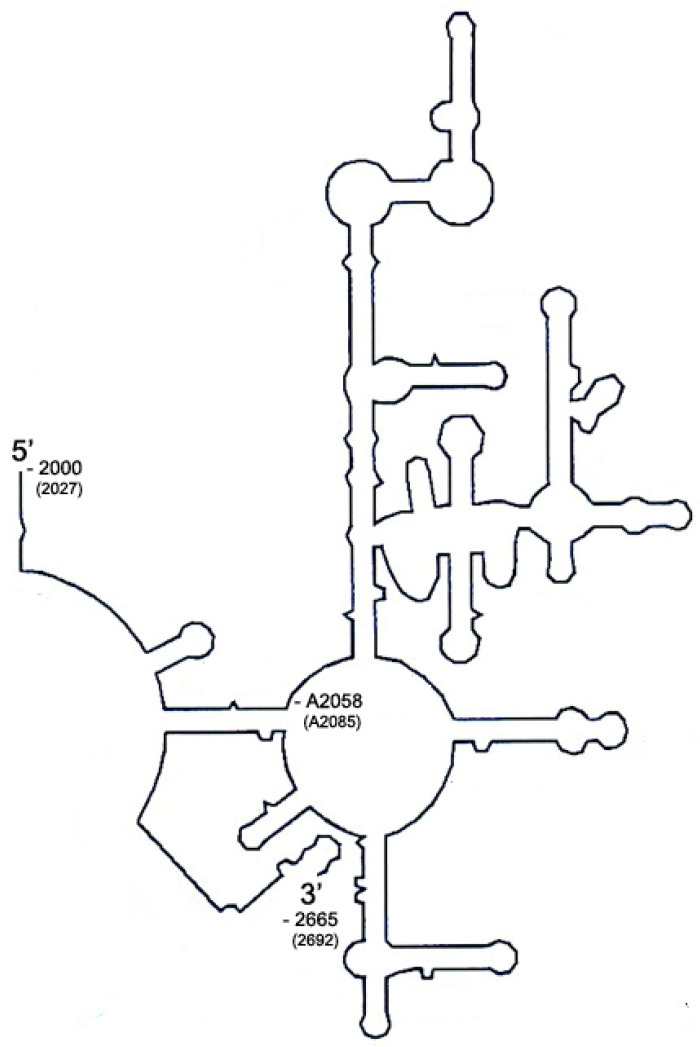
Schematic diagram of domain V used in this study. Domain V used in this study originates from *Bacillus subtilis* and consists of 666 nucleotides starting from C2000 (*E. coli* coordinate) to A2665 with *B. subtilis* coordinate number in parenthesis. The target adenine of Erm proteins is located at A2058 (A2085). In vitro transcribed RNA contains additional two nucleotides derived from *Bam*HI recognition site introduced for cloning and used for run-off transcription.

**Figure 4:**
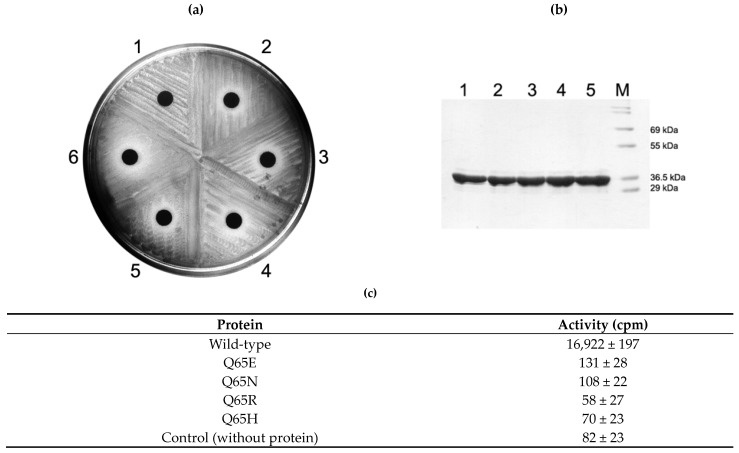
In vivo and in vitro analysis of Q65 mutant protein activity. Although all mutant proteins of Q65 could be overexpressed in *E. coli* in a similar amount and successfully purified almost equally (**b**), they could not confer resistance to erythromycin on the cells expressing it (**a**) and lost its methylating activity in vitro (**c**). See Discussion for details. 1, *E. coli* BL21(DE3) expressing the wild-type protein in (**a**) and its purified protein in (**b**); 2, cells expressing Q65E mutant and its purified protein; 3, cells expressing Q65N mutant and its purified protein; 4, cells expressing Q65R mutant and its purified protein; 5, cells expressing Q65H mutant and its purified protein; 6, cells containing only empty vector, pET23b; M, molecular weight marker.

**Figure 5:**
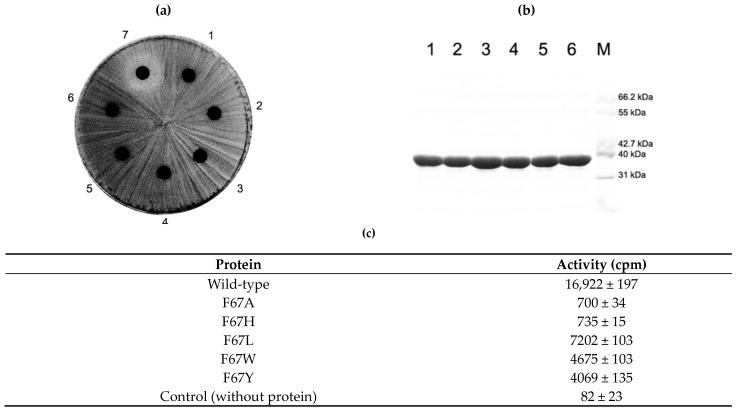
In vivo and in vitro analysis of F67 mutant protein activity. Like other mutant proteins investigated in this study, the constructed mutant proteins of F67 could be overexpressed in *E. coli* and purified in a similar amount (**b**). Whereas all mutant proteins retained the capability to confer resistance to erythromycin on the cells expressing it (**a**), the in vitro methyl group transferring activity varied (**c**). The introduction of the smaller side chain (F67A) and/or probable positive charge (F67H) could cause a huge decrease in its activity. With still a smaller but increased side chain (F67L), the reduction in activity could be alleviated. However, a bigger side chain than phenylalanine could deteriorate the situation resulting in a large reduction in activity (F67W and F67Y). See Discussion for details. 1, *E. coli* BL21(DE3) expressing wild type protein in (**a**) and its purified protein in (**b**); 2, cells expressing F67A mutant and its purified protein; 3, cells with F67H and its purified protein; 4, cells with F67L and its purified protein; 5, cells with F67W and its protein; 6, cells with F67Y and its protein; 7, cells containing only empty vector, pET23b; M, molecular weight marker.

When amino acids (threonine, phenylalanine and tyrosine) that harbor larger side chains than serine were introduced, the activity to induce the resistance against erythromycin in the cell disappeared. This observation could be verified with the in vitro activity of purified proteins (Figure 2b,c). However, the enzymatic activities of mutants that contain similar or smaller side chains than S64 protected the cell and exhibited no inhibition zone around the disc containing 200 µg erythromycin which is the proper amount of antibiotic, with that *E. coli* cell expressing mutant Erm protein without activity or no Erm could produce the reasonably measurable inhibition zone (Figure 2a, Figure 4a, Figures 4a and 5a). To further characterize the mutants of S64 that conferred resistance to erythromycin by the retained MTase activity, the in vitro activity of each mutant toward domain V, a complete substrate that has been known to contain all the structural elements for Erm proteins [35,36] (Figure 3) was measured. Each mutant, S64G, S64A and S64C exhibited 71%, 21%, and 20% activity compared to the wild-type enzyme (Figure 2c). 

### 2.4. Activity of Q65 Mutants

When Q65N mutation was introduced in ErmS and expressed in *E. coli*, resistance to erythromycin was lost and the susceptible phenotype was expressed. Besides this, cells expressing any other mutants constructed in this study, such as Q65E, Q65R, and Q65H, also formed the inhibition zone around the disc containing 200 µg erythromycin. Even purified mutant proteins appeared to lose their methyl group transferring activity to domain V (Figure 4). 

### 2.5. Activity of F67 Mutants 

In contrast to Q65 mutants, all introduced mutations for F67, whether conservative or nonconservative, appeared to render the methyl group transferring activity in the cell to confer resistance to erythromycin. However, the in vitro methyl group transferring activity varied depending on what side chain is present instead of the phenylmethyl group of phenylalanine. When the size of the side chain is reduced to alanine or presumably introduces a positive charge, the imidazole ring of histidine could decrease the methyl group transferring capability to about 4%. However, increasing the side chain from methyl (alanine) to isobutyl (leucine) promoted the activity more than 10 times those of F67A and F67H. The introduction of a larger side chain than phenylalanine (W and Y) could reduce the activity to about one-fourth of the wild-type enzyme activity (Figure 5). 

## 3. Discussion

**Figure 6:**
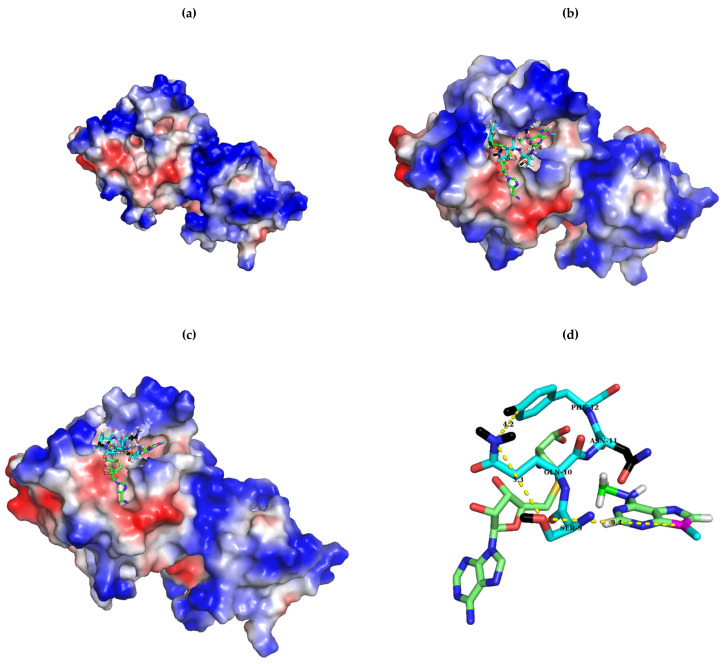
Relative positions in the ErmC’ structure of SAH, the target adenine, and the catalytically important residues investigated in this study, S9, Q10 and F12. As shown in the surface structure of the apo-form of ErmC’ ((**a**); PDB ID: 1QAM), two pockets are well developed for cofactor (SAM; left) and the target adenine (right). Whereas the structure of bound SAH (PDB ID: 1QAN) was obtained experimentally with X-ray crystallography, the target adenine was docked into its pocket [13] (The docked structure with the target adenine was a gift from Abbott Laboratories.) For SAM and the target adenine, C, O, N, and H are colored green, red, blue, and white, except for N^9^ of the target adenine and the bonded carbon to it, which is magenta and cyan, respectively. For S–Q–F, C is colored cyan to be recognized more easily, but the other atoms are the same colors as above. Note that the sulfur of homocysteine is colored yellow. Hydrogens of the surface-exposed group of the side chain of S–Q–F and the carbons in side chain of N11 are colored black for easy recognition. It is noteworthy that the surface-exposed groups of the S–Q–F side chain are located close to each other, covering the outside SAM pocket (**b**), whereas that of N is on the opposite side to form the upper inside part for the target adenine binding pocket (**c**). They form a group of side chains distancing 5.3 Å between oxygen of serine and carbamoyl nitrogen of Q and 4.2 Å between nitrogen of the carbamoyl group of Q and C3 of the phenyl group of F. For simplicity, only 9SQNF12, SAM, and the target adenine are shown in (**d**) but in different orientation. Images were generated using PyMOL.

When all Erm proteins known to date were aligned (Figure 1), the length of the sequence from the N-terminal up to the shortest motif X, which is represented by GQNF, constituting the NTER of the protein varied in a great range from 6 amino acids for Erm49 to 63 amino acids for ErmS. Furthermore, any recognizable consensus sequence could not be assigned among them. But just after this, a conserved sequence could be detected in Erm proteins as the shortest motif X ever considered, in a relaxed form (G/S/T)-Q-(N/H)-(F/L/Y) if Erm37 and Erm41 are excluded. In ErmS, this sequence is expressed as 64SQNF67 (Figure 1). In ErmS, S64T, F and Y mutations could not confer resistance to erythromycin on the cells expressing each mutant protein, presumably due to the abolished methyl group transferring activity that could not be observed in vitro either. From this observation, a conclusion could be drawn that a larger side chain than S might hamper the proper functioning of S64 even with T which contains one more additional methyl group than serine. However, in some Erm proteins such as ErmA, Erm43, Erm45 and Erm49, T could be located at this position. Probably, ErmS harbors the longest NTER that contains many arginines (16 R of 63 amino acids) and has been observed to interact with substrate RNA (H. J. Lee and H. J. Jin, unpublished results) and might require more compact interaction around this area, not allowing extra methyl group for proper activity. When the size of the side chain was similarly maintained, such as S64C, or reduced such as S64G and S64A, resistance could be observed. Employing domain V as substrate, about 71%, 21%, and 20% activity in each mutant, S64G, S64A and S64C could be recovered, respectively. From the in vitro methyl group transferring activity of each mutant of ErmS, the function of this residue could be inferred with the help of bioinformatics analysis. In more than half of the Erm proteins, glycine positions itself at this site: 26 Erms out of 44. The least activity was lost with S64G mutation among the mutants studied, consistent with conservation in Erm proteins. However, a huge amount of activity (~80% compared to the wild-type enzyme) was lost to a quite similar extent with S64C and S64A (Figure 2c), suggesting that once certain interactions occurred, the appropriate size of the side chain (hydroxymethyl vs. methyl) and the exact electronegativity and/or acidity and/or even the size of an atom (oxygen vs. sulfur) might be required because the change of oxygen to sulfur could not be properly tolerated. From this observation and the fact that threonine is present in some Erms, the hydroxyl group might be considered to be important at this position for the interaction with the substrate (see below). No cysteine and alanine could be found at this position in any Erm protein discovered up to date. For Q65, all Erm proteins possess glutamine at that position, except Erm37 and Erm41, where W is located instead of Q. The Q65N mutant in which only the methylene group was shortened from Q65 lost the methyl group transferring activity to protect the cell from erythromycin and in vitro. Q65E in which isosteric change occurred lost the ability to confer resistance on the cells expressing it and could not transfer the methyl group to domain V in vitro as well. Furthermore, Q65R and Q65H could not protect the cells from the action of erythromycin and lost their activity in vitro. Therefore, the carbamoyl group with the proper distance from the main chain of protein might be important. This might be vital to the methyl group transferring activity of the enzyme because Q exhibits almost definite conservation among Erm proteins and it appears to be permissive only to Q. In Erm37 and Erm41, Q is replaced by W. Both of them do not harbor the C-terminal domain, and Erm37 exhibits promiscuous activity [28] and Erm41 shows lethargic activity [29]. F67 is conserved quite well. It could be found among 38 out of 44 Erm proteins except Erm37, Erm41, ErmK, ErmD, Erm50 and Erm51. In Erm37, F is substituted with R; in Erm41, P is located at this position instead of F. In both ErmK and ErmD, F is more conservatively substituted with L; in Erm50 and Erm51, Y is present at that position. When F67L mutation was introduced in ErmS, the methyl group transferring activity was reduced to 42% compared to the wild-type protein, but the retention rate of activity is the highest among the mutant enzymes studied, being well corroborated by the conservation with ErmK and ErmD. The further reduction of the side chain to methyl—as in alanine—or the probable introduction of the positive charge in the side chain—such as histidine—reduced the activity to about 4% of the wild-type. The larger side chains of tyrosine and tryptophan in place of F decreased the activity to less than that of F67L, exhibiting about one-fourth of the wild-type enzyme, indicating that hydrophobicity with proper size might be required at this position, although Erm50 and Erm51 allow a bigger side chain by hydroxyl group: hydroxyphenyl to phenyl. Therefore, the absolute and/or relatively strict requirement on the identity of the side chain in this shortest motif X should be met for its appropriate activity. These observations might suggest some or all of these amino acids studied interacting with something else other than SAM, RNA, although they are part of motif X, or motif X itself, which interacts with SAM. The fact that a range of lengths of amino acids could be defined for motif X and four amino acids investigated in this study constitute the well conserved shortest one (see below) might mean that the shortest motif X may be associated with the core function of motif X or other functions unique to the Erm family. Although motif X, one of the nine common SAM-dependent MTase functional motifs, motifs I to VIII and X observed in amino (N^4^-cytosine or N^6^-adenine) MTases [18] is well known to be located before motif I in Erm and KsgA/Dim1, its identity, especially the range of sequence encompassing motif X, is different depending on the report. It ranged from 18 amino acids [19], 6 amino acids [37] to 4 amino acids [17] (GQNF is the most appeared amino acids). Residue V21 in M^.^TaqI, a member of motif X and its counterpart in KsgA from Aquifex aeolicus, H11 may play multiple roles. Especially, H11 was suggested to function by interacting with both the cofactor and the target adenine and linking two catalytically important motifs, motifs IV and VII [37]. Therefore, the shortest motif X could take part in more than just one function. Furthermore, in the bound structure of ErmC’ with SAH and docked target adenine, the side chains of these amino acids (S9, Q10 and F12 as S64, Q65 and F67 in ErmS) do not interact with SAM pointing outward to face the solvent, noticeably the hydroxymethyl group of S9, the amine of the carbamoyl group of Q10, and 3, 4, and 5 positions of the benzene ring in the phenylmethyl group of F12 (Figure 6b,c). In that complex structure, side chains of S9, Q10, and F12 are closely located together and form the outer surface of the SAM binding pocket, whereas roughly their main chains form part of the inside wall of the SAM binding pocket: The distances between the nitrogen of the carbamoyl group of Q10 and the oxygen of the hydroxyl group of S9 is 5.3 and 4.2 Å between carbamoyl nitrogen and C4 carbon of the phenyl group of F12 (Figure 6b–d). They might contribute to the binding to the substrate RNA together. However, the side chain of N11 participates in forming the upper inside part of the wall of the methylatable adenine binding pocket and is suggested to be involved in the stabilization of the target adenine [20]. Actually, S9-Q10-F12 and N11 are sequentially adjacent in the primary structure, but their side chains are located on the opposite sides in the tertiary structure (Figure 6c).

**Figure 7:**
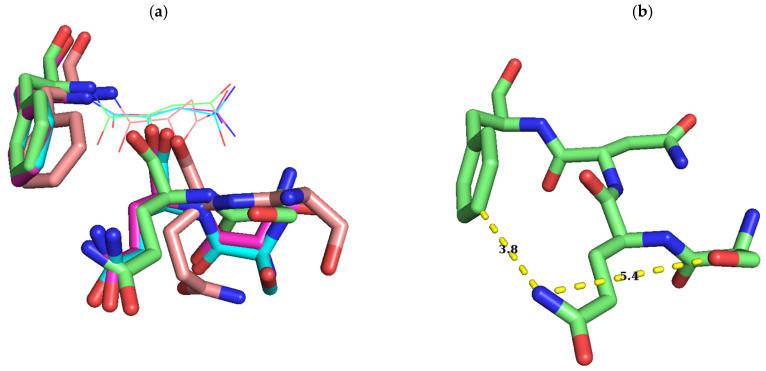

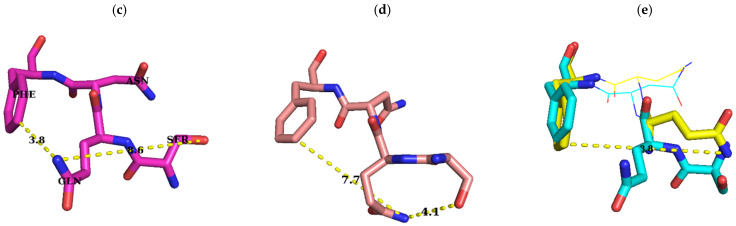
Conformational change of S–Q–F residues with binding to different cofactors. Conformations of S–Q–F residues in ErmC’ structures which are bound with SAM (PDB ID: 1QAO), SAH, and sinefungin (PDB ID: 1QAQ) and docked with the target adenine [13] and apo-form (only Q and F). Whereas O and N are colored red and blue, C has different colors: green, the conformation bound with SAM (b); magenta, the one with SAH (**c**); orange, the one with sinefungin (**d**); cyan, that docked with the target adenine as above in Figure 6. The structure docked with the target adenine was originated from the structure complexed with SAH and the conformations of this region in two structures are quite similar to each other, except S9, presumably affected by the docked target adenine (**a**). After being docked by the target adenine, the distance between nitrogen of the carbamoyl group of Q10 and oxygen of the hydroxyl group of S9 is shortened as seen in (**c**) and (**d**) in Figure 6, and in (**c**) in this Figure 7. Conformations of S–Q–F with the SAM-bound structure are modestly different from those of SAH, with S9 being quite distinct. However, on binding with sinefungin, S9 and Q10 exhibit a big difference in conformation but with moderate change in F12 (a). While Q10 has a slight conformation change with the binding of SAM and SAH, and after docking with the target adenine, a huge conformational change in this residue occurs on shift from the apo-form (yellow C) to one of these structures reducing the distance between nitrogen of the carbamoyl group of Q10 and C3 of the phenyl group of F12 (**e**). Although nitrogen of the carbamoyl group of Q10 of complexed structures and the apo-form points to almost opposite direction, they are facing outward and surface-exposed. All these observations might support the notion that SQNF, the shortest motif X, is intrinsically flexible presumably along with the NTER. Distances between C3 of phenyl group of F12, and nitrogen of carbamoyl group of Q10, and oxygen of hydroxyl group of S9 and nitrogen of carbamoyl group of Q10 is shown in Å for easy recognition of conformational change. Residue name is designated in (**c**). Images were generated using PyMOL.

Even in the apo form of the enzyme, the side chains of Q10 and F12 being further separated than the complexed structure are directed away from SAM to face the solvent with S9 disordered (Figure 7e). These residues are located immediately next to the bound SAM within a distance of 6 Å, forming a part of the binding pocket for SAM and only region of the protein where conformation was changed with binding to different ligands, SAM, SAH, and sinefungin, exhibiting the intrinsic flexibility (Figure 7a–d) [13] presumably to allow fitness for binding to substrate, RNA. The structures of the apo-form (visible including Q10 but not S9) and enzyme bound with three ligands (visible with S9) are quite similar, except three segments: N terminus with different electron densities and huge different conformations of Q10, modest conformational difference of F12, and other two segments related to the amino acids directly interacting with the ligands [13].

Whether it is bound to ligands, ErmC’ appeared to develop the similar size and structure of pockets for ligand and the methylatable adenine to those observed in ligand bound enzyme and its docked structure with the methylatable adenine (Figure 6a), presumably supporting the random bi-bi reaction mechanism [38]. Although a few distinct reaction mechanisms for biological SAM-dependent methyl transfer reaction have been proposed [39], there has been a strong consensus that biological methylation in a specific adenine by Erm proteins might occur by the conventional S_N_2 reaction mechanism [13]. It has been well known that for proper catalysis, it is most critical to maintain the optimal orientation of SAM and the target base rather than to stabilize SAM and the target base when this mechanism is applied [40,41]. In the structures of ErmC’, the hydroxymethyl group of S64, carbamoyl group of Q65 and phenyl group of F67 in the side chain of each amino acid form a group of side chains (Figure 7a) facing the solvent in close vicinity, and at those positions, the requirement of side chain identity for proper catalysis is very strict, as described above. In the case of Q65, its requirement is almost absolute for the activity. In Erm proteins, the region from the N-terminal end to this region might exhibit high intrinsic flexibility, which might contain a presumed disordered NTER as in ErmC’. Recently, the disordered regions of RNA binding proteins have been shown to have a potential for highly selective interaction with a defined target sequence or of structural requirements. Moreover, this flexible NTER, including S–Q–F, might cooperate with the highly conserved Rossman fold domain to achieve the specific and selective interaction and increase the affinity [42,43]. Furthermore, S–Q–F locates itself quite close to the methylatable adenine distancing 9.4 Å between N^9^ of the target adenine and side chain oxygen of S9 in the ErmC’ structure (Figure 6b–d). All these observations strongly suggested that S–Q–F collectively interacts with part of substrate RNA close to the methylatable adenine and might contribute to maintaining the optimal orientation between the transferrable methyl group of SAM and the amine group of the target adenine, with Q performing a pivotal role. Previously, it has been reported that the active site of rRNA:m6A MTase of ErmC’ tolerated amino acid substitutions much more well than that of DNA:m6A MTase [20] possibly implying that the interaction for the optimal orientation might be further provided outside the active site because even in the RNA methylation system, proper orientation between the methyl group of the cofactor, SAM and the target adenine should be still necessary for the proper catalysis. Presumably this could be caused by the more flexibility of RNA than that of DNA [17]. 

Although not a few water molecules could be located inside the binding pockets of SAM and the methylatable adenine in the crystal structure of apo enzyme of ErmC’, in the active site, probably water should be removed before the reaction takes place because water could form the hydrogen bond(s) with the reactive lone pairs on the nucleophile, exocyclic amino group of adenine, or depending on the environment in the active site of enzyme, water might act as nucleophile instead of the amino group of adenine. The exclusion of water could reduce the polarity in the active site and facilitate the nucleophilic reaction. The exclusion of water molecules probably together with binding of both substrate RNA and cofactor could induce the conformational change of the enzyme, which might include the blockage of the entrance of the binding pockets to prevent any involvement of water molecules that haphazardly come in, at least during catalysis by the S_N_2 reaction. This occlusion presumably may occur mostly by the NTER amino acids before or including the 64SQNF67 with the help of other amino acids in the protein, including the pocket forming amino acids. It could be conceived that this conformational change of protein could be accompanied with that of RNA substrate because the previous complex structure based on the amino acid mutagenesis study and restriction of protein conformation could not predict the interaction between SQNF in ErmC’ and RNA and their distance was too far to make contact [15]. Because each Erm protein structure complexed with substrate RNA and cofactor or at least one of them is not available now, it is not possible to exactly delineate the local structure of this region formed by being bound with RNA and cofactor. However, there is much variation in length and identities of amino acids constituting this region (NTER). This region or part of it could contribute to the shifting of substrate specificity from that of KsgA to Erm’s [17], presumably implying that this region represents unique substrate binding mode of Erm proteins and might take conformations unique to Erm protein(s) accordingly. Therefore, the Erm protein might assume a distinct local structure with bound substrate RNA in this region and provide the target site to develop the unique inhibitor(s) for enhanced selectivity and presumably with high potency. Furthermore, there is no other perceived homolog, except for KsgA/Dim family proteins [16] that utilize somewhat different substrates with unique structure and topology for recognition and methylation [44,45,46], and Erm proteins harbor distinct sequence and length of the NTER. Therefore, there could not be cross-reactivity to induce toxicity when inhibitors to be developed are administered, although the KsgA/Dim family appears to exhibit some sequence conservation around the shortest motif X with the Erm protein family. Therefore, this region, including the shortest motif X, GQNF bound with RNA substrate, might be a potential target site for developing inhibitors for Erm proteins, which could be co-administered with MLS_B_ antibiotics to escape the resistance caused by this formidable antibiotic resistance factor protein, Erm.

## 4. Materials and Methods

### 4.1. Materials

*E. coli* DH5α (Promega, Madison, WI, USA) and BL21(DE3) (Novagen, Madison, WI, USA) were used for general cloning and expression of His_6_-tagged Erm proteins, respectively. The sequence for domain V of 23S rRNA used in this study was cloned from *Bacillus subtilis* BD170 and exhibited three sequence differences compared with the one presented on the Gutell Lab’s Comparative RNA Website (CRW site: http://www.rna.icmb.utexas.edu, accessed on 3 December 2020), including two mutations (C2203G and U2629A) and one nucleotide deletion (Δ C2473). While 19 identical sequences were identified upon a search of GenBank with our sequence as a query, only 2 sequences showed exact sequence identity with the one from the Gutell Lab’s CRW site. Restriction endonucleases were purchased from New England BioLabs (Beverly, MA, USA) and used as recommended by the supplier’s manual. LB media and Bacto agar for bacterial culture were from Difco Laboratories (Detroit, MI, USA). For PCR, Taq polymerase and nucleotides were obtained from TaKaRa Shuzo Co. (Otsu, Shiga, Japan). For in vitro transcription, spermine, Triton X-100, and polyethylene glycol (PEG; molecular weight, 8000) were obtained from Sigma Chemical Co. (St. Louis, MO, USA), and nucleotides were obtained from TaKaRa Shuzo Co. The T7 RNA polymerase was prepared “in-house.” The His·Bind resin was from Novagen. Reagents for polyacrylamide gel electrophoresis, such as acrylamide, bis-acrylamide, ammonium persulfate, and *N*,*N*,*N*′,*N*′-tetramethylethylenediamine (TEMED), were obtained from Bio-Rad (Hercules, CA, USA). Most of the conventional chemicals, such as salts, buffer components, agarose, and antibiotics, were purchased from Sigma Chemical Co.

#### 4.1.1. Site-Directed Mutagenesis and Construction of Expression Vector

The expression vector (pHJJ105) and *E. coli* strain (*E. coli* HJJ105) overexpressing the wild-type ErmS was obtained in previous studies [25,34]. Site-directed mutagenesis was carried out by a sequential PCR method designated overlap extension PCR [47]. To obtain the DNA fragment for S64A covering the 5′-end region, PCR was performed using the pHJJ105 plasmid DNA as the template and the oligonucleotides oligo-1 and oligo-4 as the forward and reverse primers, respectively. To obtain the DNA fragment for S64A covering the 3′-end region, PCR was performed using the oligonucleotides oligo-3 and oligo-2 as the forward and reverse primers, respectively. In the next PCR, two DNA fragments obtained above, covering the 5′- and 3′-end regions that contain the overlapping region harboring the mutated site (S64A) in common, were combined along with the two oligonucleotides, oligo-1 and oligo-2, forward and reverse primers. The reaction was carried out to obtain the whole *ermS* gene harboring the S64A mutation. The sequences of primers were modified to include a restriction site for *Nde*I (5′-catatg-3′), overlapping the initiation Met codon (oligo-1), and a site for *Hin*dIII (5′-aagcct-3′, oligo-2). The resultant PCR product was directly digested with *Nde*I and *Hin*dIII restriction enzymes, and the DNA fragment containing the S64A mutation was ligated into pET23b *Nde*I-*Hin*dIII sites. The cloned gene was sequenced to confirm the sequence and frame of the insert. Construction of expression vectors for all the other mutant genes was performed in the same way. All the primers for cloning of mutant *ermS*-encoding DNA fragments are summarized in Table S1.

#### 4.1.2. In Vivo Activity Assay for ErmS and Its Mutant Proteins (Antibiotic Susceptibility Assay) 

8 µL of erythromycin stock solution (25 mg/mL) was dropped onto Whatmann 3MM paper punched to have circular shape to reach the final amount of 200 µg and dried in the air. Well-grown *E. coli* cultures were transferred to new LB medium (10%, *v*/*v*) and incubated at 37 °C for another 1.5 h to reach an A_600_ of 0.8–1.0, and then spread on pre-warmed LB agar plate with cotton swabs. To test for antibiotic resistance, the dried circle papers containing erythromycin were placed in the center of the susceptible (harboring pET23b), resistant (harboring pHJJ105) and testing (expressing mutant proteins) cultures. They were incubated overnight at 37 °C and the inhibition zone was examined, which was formed due to retarded growth by the inhibitory action of erythromycin.

#### 4.1.3. Protein Expression and Purification

Transformed *E. coli* BL21(DE3) cells with wild type *ermS* or mutant *ermS genes* in the pET-23b vector were grown overnight at 37 °C in LB medium supplemented with 100 mg/mL of ampicillin. Those were transferred to new LB medium (10%, *v*/*v*) and incubated at 37 °C for another 1.5 h to reach an A_600_ of 0.8–1.0. In order to induce the expression, IPTG (isopropyl-β-d-thiogalactopyranoside) was added to the final concentration of 1 mM and incubation continued for another 18 h at 22 °C. Purification was performed using previously described procedure [45]. Briefly, cells from 100 mL of culture were collected by centrifugation and resuspended in buffer A (20 mM Tris-HCl [pH 7.0], 500 mM KCl, and 5 mM imidazole). The cells were disrupted by sonication on ice using a GEX-130 ultrasonic processor (130 W, 20 kHz) at 50% amplitude for 5-s pulses with 10-s pauses for cooling. The total sonication time was 5 min. The lysate was centrifuged to remove the cell debris and other insoluble materials, including inclusion bodies, and the supernatant was loaded onto a column containing His·Bind resin preequilibrated with buffer A. Next, the column was washed extensively with buffer B (20 mM Tris-HCl [pH 7.0], 500 mM KCl, and 100 mM imidazole) to remove unbound and falsely bound proteins, and proteins were eluted with buffer C (20 mM Tris-HCl [pH 7.0], 500 mM KCl, and 300 mM imidazole). To remove the imidazole and salt, the eluted protein solution was purified using a PD-10 desalting column, as described by GE Healthcare (Little Chalfont, Buckinghamshire, UK) and was stored at −20 °C in 20 mM Tris-HCl (pH 7.0), 200 mM KCl, 1 mM EDTA, and 50% glycerol. The protein concentration was determined by the bicinchoninic acid (BCA) protein assay method (Pierce, Rockford, IL, USA).

#### 4.1.4. Cloning of *B. subtilis* Domain V DNA and Its In Vitro Transcription

To obtain the DNA fragment encoding domain V, PCR was performed using a *B. subtilis* BD170 chromosomal DNA as a template and two oligonucleotides GGAATTCtaatacgactcactataGAGAGACT CGGTGAAATTATAG and CGGGATCCTCTCGTACTAAGGACAGCTC as forward and reverse primers, respectively. The sequences of these primers correspond to nucleotides 2000 to 2021 and 2643 to 2665 in *E. coli* coordinate (2027 to 2048 and 2670 to 2692 in *B. subtilis* coordinate). The underlined sequences in oligonucleotides introduced *Eco*RI and *Bam*HI restriction sites, respectively. Lowercase nucleotides indicate the T7 promoter sequence. The resultant PCR product was directly digested with *Eco*RI and *Bam*HI restriction enzymes, and the DNA fragment containing the domain V gene was ligated into pUC19 *Eco*RI and *Bam*HI sites. The cloned gene was sequenced to confirm the sequence and frame of the insert. Domain V was transcribed in vitro using phage T7 RNA polymerase. The plasmid was used as the templates for in vitro transcription. The plasmid was linearized with *Bam*HI for runoff transcription. After digestion with *Bam*HI, five nucleotide sequences should be provided by the overhang from digestion, three of which were exactly the same as the sequence of domain V. Therefore, in the final transcript produced, two nucleotides (UC) derived from *Bam*HI recognition sequence are additionally provided (668 nt; Figure 2). The linearized plasmid was used directly as the template for the synthesis of substrate RNA transcripts. Transcription from the linearized plasmid templates was performed in 500-μL reaction mixtures containing 40 mM Tris-HCl (pH 8.1), 5 mM dithiothreitol (DTT), 1 mM spermine, 0.01% Triton X-100, 80 mg/mL PEG, 25 μg of DNA template, 4 mM ribonucleoside triphosphates (rNTPs), 28 mM MgCl_2_, and 10 μg of T7 RNA polymerase (prepared in-house) at 37 °C for 4 h. After transcription, the transcripts were extracted with phenol-chloroform, precipitated with ethanol, and resuspended in diethyl pyrocarbonate (DEPC)-treated water. The transcripts produced were visualized with UV on a 5% 7 M urea-polyacrylamide gel to check the integrity and verify the size. The band with the correct size was extracted in a Tris-borate-EDTA (TBE) buffer by electrophoresis; and after ethanol precipitation, it was dissolved in self-folding buffer (50 mM HEPES-KOH [pH 7.5], 20 mM magnesium acetate, and 400 mM NH_4_Cl), heated to 65 °C for 10 min, and then cooled to 37 °C over a period of 90 min to complete the self-folding of RNA transcripts.

#### 4.1.5. In Vitro Methylation Assay

In vitro methylation of BDV (*B. subtilis* 23S rRNA domain V, 668 nt) substrates by Erm proteins was carried out by a slightly modified version of a previously described procedure [34,35,48]. The reaction was performed in 50 μL volumes containing 50 mM Tris-HCl (pH 7.5), 4 mM MgCl_2_, 40 mM KCl, 10 mM dithiothreitol, 3.3 pmol S-adenosyl-l-methionine (SAM; specific activity, 80 Ci/mmol; PerkinElmer), 10 pmol rRNA transcripts, and 6.76 pmol (250 ng) purified Erm proteins. Reaction mixtures containing everything except proteins were prewarmed to 37 °C by at least 5 min of incubation, and then purified Erm proteins were added to prewarmed tubes to minimize any lag in the start of the reaction. After 1 h incubation, ice cold 0.5 mL of 12% trichloroacetic acid was added in order to terminate the reaction. The methylated RNAs collected by centrifugation were washed twice with 1.25 mL of ice-cold 6% trichloroacetic acid. After drying, the precipitate was extracted with 3 mL of scintillation fluid (Ultima Gold; Packard), transferred to a counting vial and counted (Tri-Carb 2900TR; Packard, Shelton, CT, USA). Experiments were repeated at least thrice. 

## Data Availability

Coordinates for the docked structure with the target adenine into ErmC’ complexed with SAH might be obtained with the permission of Abbott Laboratories.

## Supplementary Materials

The following are available online at https://www.mdpi.com/2079-6382/10/3/264/s1, Table S1: DNA oligonucleotides used in cloning DNA fragments encoding various *ermS* mutants.
