# Supplementary material for: Potential Target Site for Inhibitors in MLSB Antibiotic Resistance
Source: Antibiotics (Basel). 2021 Mar 5;10(3):264. doi: 10.3390/antibiotics10030264 (PMC7998614; doi:10.3390/antibiotics10030264)
Supplement: Supplementary file 1 [file antibiotics-10-00264-s001.pdf]

Supplementary Table 1. DNA oligonucleotides used in cloning DNA fragments encoding various *ermS* mutants.

Deoxyoligo - nucleotide	Sequence (5′ → 3′)	Description
Oligo-1	GGAATTC**CATATG**GCTCGTGCAC-CGCGTTCT	31-mer forward primer for *ermS* cloning, which contains restriction enzyme site overlapping the initiation Met codon (*Nde*I, bold)
Oligo-2	CCC**AA-GCTT**CCGTCCGGCCGGTCGGCT	27-mer reverse primer for *ermS* cloning which contains restriction enzyme site (*Hind*III, bold)
Oligo-3	**CGCGAGCTCGCTCAGAACTTC**CTCGCC	27-mer forward primer for S64A cloning
Oligo-4	**GAAGTTCTGAGCGAGCTCGCG**CCGCGC	27-mer reverse primer for S64A cloning
Oligo-5	**CGCGAGCTCTGCCAGAACTTC**CTCGCC	27-mer forward primer for S64C cloning
Oligo-6	**GAAGTTCTGG-CAGAGCTCGCG**CCGCGC	27-mer reverse primer for S64C cloning
Oligo-7	**CGCGAGCTCGGTCAGAACTTC**CTCGCC	27-mer forward primer for S64G cloning
Oligo-8	**GAAGTTCTGAC-CGAGCTCGCG**CCGCGC	27-mer reverse primer for S64G cloning
Oligo-9	**CGCGAGCTCTTCCAGAACTTC**CTCGCC	27-mer forward primer for S64F cloning
Oligo-10	**GAAGTTCTGGAA-GAGCTCGCG**CCGCGC	27-mer reverse primer for S64F cloning
Oligo-11	**CGCGAGCTCACCCAGAACTTC**CTCGCC	27-mer forward primer for S64T cloning
Oligo-12	**GAAGTTCTGGGTGAGCTCGCG**CCGCGC	27-mer reverse primer for S64T cloning
Oligo-13	**CGCGAGCTCTAC-CAGAACTTC**CTCGCC	27-mer forward primer for S64Y cloning
Oligo-14	**GAAGTTCTGG-TAGAGCTCGCG**CCGCGC	27-mer reverse primer for S64Y cloning
Oligo-15	**GAGCTCTCG-GAAAACTTCCTC**GCCCGC	27-mer forward primer for Q65E cloning
Oligo-16	**GAGGAAGTTTTCCGA-GAGCTC**GCGCCG	27-mer reverse primer for Q65E cloning
Oligo-17	*GAGCTCTCG*AAC*AACTTCCTC*GCCCGC	27-mer forward primer for Q65N cloning
Oligo-18	*GAGGAAGTT*GTT*CGA-GAGCTC*GCGCCG	27-mer reverse primer for Q65N cloning
Oligo-19	*GAGCTCTCG*A-GA*AACTTCCTC*GCCCGC	27-mer forward primer for Q65R cloning
Oligo-20	**GAGGAAGTTTCTCGA-GAGCTC**GCGCCG	27-mer reverse primer for Q65R cloning
Oligo-21	**GAGCTCTCG-CACAACTTCCTC**GCCCGC	27-mer forward primer for Q65H cloning
Oligo-22	**GAGGAAGTTGTGCGA-GAGCTC**GCGCCG	27-mer reverse primer for Q65H cloning
Oligo-23	**TCGCAGAACGCAC-TCGCCCGC**CGGGCC	27-mer forward primer for F67A cloning
Oligo-24	**GCGGGCGAGTGCGTTCTGCGA-**GAGCTC	27-mer reverse primer for F67A cloning

Oligo-25	**TCGCAGAACCAC-CTCGCCCGC**CGGGCC	27-mer forward primer for F67H cloning
Oligo-26	**GCGGGCGAGGTGGTTCTGCGA-**GAGCTC	27-mer reverse primer for F67H cloning
Oligo-27	**TCGCAGAAC-CTGCTCGCCCGC**CGGGCC	27-mer forward primer for F67L cloning
Oligo-28	**GCGGGCGAGCAGGTTCTGCGA-**GAGCTC	27-mer reverse primer for F67L cloning
Oligo-29	**TCG-CAGAACTGGCTCGCCCGC**CGGGCC	27-mer forward primer for F67W cloning
Oligo-30	**GCGGGCGAGCCAGTTCTGCGA-**GAGCTC	27-mer reverse primer for F67W cloning
Oligo-31	**TCGCAGAACTAC-CTCGCCCGC**CGGGCC	27-mer forward primer for F67Y cloning
Oligo-32	**GCGGGCGAGGTAGTTCTGCGA-**GAGCTC	27-mer reverse primer for F67Y cloning

Note. To obtain the mutant *ermS* gene by overlap extension PCR, four oligonucleotides are necessary. While two nucleotides cover the N- and C-terminal ends, the other two cover the middle region containing the site to be mutated. The oligonucleotides covering middle region should contain the overlapping region to be extended and amplified by oligonucleotides covering the N- and C-terminal ends (oligo-1 and oligo-2). The underlined sequence in oligo-3 to oligo-32 denotes the codon sequences and its base-pairing sequences introduced for mutagenesis. The bold sequences in oligo-3 to oligo-32 represent the overlapping sequence for extension.
